# Supplementary material for: Transferability of genetic loci and polygenic scores for cardiometabolic traits in British Pakistani and Bangladeshi individuals
Source: Nat Commun. 2022 Aug 9;13:4664. doi: 10.1038/s41467-022-32095-5 (PMC9363492; doi:10.1038/s41467-022-32095-5)
Supplement: Supplementary file 1 — Supplementary Information [file 41467_2022_32095_MOESM1_ESM.pdf]

Supplementary Information

Supplementary Methods……………………………………………………………………1

Supplementary Figure 1–15……………………………………………………………11

Genes & Health Research team members………………………………………….. 26

Supplementary References…………………………………………………………… 26

Supplementary Methods

Quality control of genotype data from Genes & Health

Quality control of genotype data was performed using Illumina's GenomeStudio and plink v1.9. We first removed variants with cluster separation scores <0.57, Gentrain score <0.7, excess of heterozygotes >0.03, or ChiTest 100 (Hardy-Weinberg test) <0.6 in GenomeStudio, as well as variants that were included on the array in order to tag specific structural variants. We removed samples with low call rate (<0.995 for male samples and <0.992 for female samples across all 637,829 variants including those on Y chromosome for males) and those that failed gender checks. When there were duplicate samples, we retained the sample with the highest call rate. Using plink, we further removed variants with low call rate (<0.99), and the variant with the lowest call rate amongst duplicate variant pairs. We excluded rare variants with minor allele frequency (MAF) <1%. The high levels of autozygosity in this cohort can cause variants to fail Hardy-Weinberg equilibrium test. We thus removed variants that failed the Hardy-Weinberg test ($p<1\times10^{-6}$) in a subset of samples with low level of autozygosity. To define these 'low-autozygosity' individuals, we pruned SNPs (LD r^2 >0.8) and called runs of

homozygosity (RoHs) using plink1.9 with default parameters, then took the 64% of the individuals who had a fraction of the genome in RoHs <0.5%.

Having identified related individuals (second degree or closer; kinship coefficient >0.0884) using KING v2.2.4[1], we performed principal component analysis (PCA) in unrelated samples, and projected the remainder onto the same PC space using smartpca from EIGENSOFT v7.2.1[2].

Identification of European-ancestry individuals from eMERGE

To identify EUR individuals from eMERGE, we performed PCA in samples from the 1000 Genomes project phase 3 dataset, and projected eMERGE participants onto the same PC space using smartpca from EIGENSOFT v7.2.1 [2]. For PCA, we restricted to LD-pruned common SNPs (MAF ≥1%) with imputation INFO score ≥0.98 in eMERGE. We identified samples that were clustered together with the EUR samples from the 1000 Genomes project using a dimension reduction method, Uniform Manifold Approximation and Projection (UMAP), applied to the first 20 PCs, performed using the R package "umap" v0.2.6.0 [3]. Self-reported Hispanic or Latino, African, Asian, American Indian or Alaska Native individuals were excluded. This resulted in 43,877 EUR individuals available for the comparison with G&H.

Phenotype and covariate definitions from electronic health-record data in Genes & Health

Of the 22,490 genotyped G&H individuals with electronic health record data, 20,830 had primary care data available through the Discovery Data Service [4] which includes clinical observations as well as current and historic diagnoses (coded using READ version 2 codes,

and recently converted to SNOMED CT codes using standard mapping protocols [5]). 17,226 had diagnosis and procedure codes (ICD10 and OPCS4 codes, respectively) extracted from the UK's largest secondary care health provider, Barts Health NHS Trust. G&H has a rich source of data on clinical diagnoses which goes back more than 20 years ago when the primary care health records were digitised around 2000, and pre-digitisation dates of diagnoses are also included [6]. We reported the proportions of the participants who had high total cholesterol and LDL cholesterol levels in the Genes & Health cohort, and the cutoffs were recommended by the UK National Health Service (https://www.nhs.uk/conditions/high-cholesterol/cholesterol-levels/).

Coronary artery disease (CAD): We defined CAD cases as those with myocardial infarction or coronary revascularization in either primary and secondary care data. We excluded individuals with angina, chronic ischemic heart disease, aneurysm or atherosclerotic cardiovascular disease from the control sample [7]. ICD10 and OPCS codes that were used to define CAD are in Supplementary Data 1. Since procedure codes were not available in eMERGE, we performed a sensitivity analysis in G&H to investigate the effects of excluding OPCS4 codes in CAD ascertainment. For this, we defined CAD solely using ICD10 codes in individuals with secondary care data, ignoring OPCS codes and primary care data; we excluded individuals without secondary care data for this analysis.

Body mass index (BMI): We used median adult height and weight measurements within the past 5 years to calculate BMI.

Adjustment of lipids for statin usage: For lipids, we took the latest adult measurements and corrected for statin usage if lipid levels were measured between the start and end date of any statin prescriptions. No adjustment was made on HDL cholesterol (HDL-C) or triglycerides. Adjustment of lipids followed the procedure in Liu *et al*. [8], as follows. To correct for statin usage, total cholesterol (TC) was replaced by TC/0.8. LDL cholesterol (LDL-C)

levels were calculated using the Friedewald equation, and statin-adjusted LDL-C was recalculated using adjusted TC levels as follows: corrected LDL-C = uncorrected LDL-C + 0.2 × adjusted TC. LDL-C/0.7 was used for 32 individuals for whom we couldn't find a TC measurement on the same date.

Adjustment of blood pressure for medication usage: We extracted the latest systolic blood pressure (SBP) and diastolic blood pressure (DBP) measurements and adjusted for blood pressure medication use by adding 15 and 10 mmHg to SBP and DBP, respectively, if the measurement coincided with any prescription date [9].

For assessing PGS accuracy, we excluded one sample in each pair of 2nd-degree relatives (kinship coefficient >0.0884 calculated using KING v2.2.4 [1]). Individuals with the highest number of relatives (and controls, if the trait is binary) were removed first. Sample sizes for each trait are in Supplementary Data 2. Quantitative traits were inverse normal transformed.

Phenotype definitions from electronic health-record data in eMERGE

We excluded participants younger than 16 years old. Lipid and blood pressure measurements were taken from dbGaP dataset phs000888.v1.p1, Data on medications affecting lipid and BP measurements were not available, so the highest measurements for LDL, TC, SBP, and DBP were used when comparing PGSs with G&H in order to minimise the effects of medications. For BMI, we took the median value from adult measurements (phs001584.v1.p1). CAD was ascertained using ICD9/10 codes which were available in the updated eMERGE Phase III dataset (phs001584.v2.p2). CAD cases and controls were defined based on secondary care ICD10 codes as described above for G&H (Supplementary Data 1).

Estimation of statistical power for replication

The pseudo R code for calculating the power at each locus is shown below [10,11].

Binary traits:

```
  alpha = replication significance level
  f = Allele frequency in controls in the replication cohort
  n = sample size of the replication cohort
  phi = n_case / n
  b = effect size in the discovery GWAS
  POWER = pchisq(qchisq(alpha, df = 1, lower = F), df = 1, ncp = 2*f*(1-
f)*n*phi*(1-phi)*b^2, lower = F)
```

Quantitative traits:

```
  f = Allele frequency in the replication cohort
  q2 = 2*f*(1-f)*b^2
  POWER = pchisq(qchisq(alpha, df = 1, lower = F), df = 1, ncp = n*q2/(1-
q2), lower = F)
```

Calculation of QRISK3 scores in Genes & Health

We used clinical data that were extracted earlier than the assessment date (1 January 2010) to calculate QRISK3. We excluded about one third of CAD cases whose diagnosis was made earlier than the assessment date (prevalent cases) and used incident cases who developed CAD later. Follow-up varied for cases and was fixed at 10 years for controls. The QRISK3 algorithm has variables that indicate whether a patient has a variety of other diseases, and these were defined using the codes shown in Supplementary Data 3, following [12]. Medication use (hypertension treatment, corticosteroid, and atypical antipsychotic medication) was defined as two or more prescriptions, with the most recent one having been issued within 28 days prior to the assessment. We used the most recent measurements taken prior to the assessment date, and kept individuals with at least three non-missing measurements out of four (height, weight, SBP, and TC). Pattern of missingness is shown in Figure S3. We kept individuals with at least three non-missing quantitative measurements out of height, weight,

SBP, HDL, and TC. Townsend index was not available in G&H, so we used the mean value (3.307) of the lowest two quintiles from the 2011 census data in the UK [13]. HDL-C levels were all measured later than 2010 in G&H, so for the TC/HDL-C ratio, we used 3.905 and 4.882 (averages calculated using later data) for females and males, respectively. To deal with missing data, we applied multiple imputation which accounts for sex, age, and genetically-defined ancestry (Bangladeshi *versus* Pakistani; identified using PCA-UMAP), using the R package "mice" v3.13.0 to impute height, weight, SBP, SD of SBP measurements within 2 years, and smoking status.

Integration of QRISK3 scores with PGS for CAD

To integrate QRISK3 scores with PGS for CAD, we followed Riveros-Mckay *et al.*[12] and calculated an integrated score by multiplying the odds converted from the QRISK3 score with the odds ratio given an individual's PGS, where the odds ratio per SD of PGS was estimated using a logistic regression in which QRISK3 and their interaction were accounted for. The logistic regression was performed in males and females separately. We used the most accurate PGS for CAD in SAS from the PGS Catalog, which was developed by Wang et al. [14]; this score was derived from EUR GWAS using LDpred and tuned in SAS individuals in UKBB. We regressed out 10 PCs from the PGS, and used the scaled residuals in the Cox regression analysis.

Sensitivity analysis to assess the potential effects of missing data on QRISK3 and the integrated score

There was a high proportion of missing data for continuous variables used in the QRISK3 algorithm (Figure S3A), especially for HDL-C which was not measured earlier than 2010 and

total cholesterols. To assess the potential effects of high missingness on the evaluation of QRISK3 and the integrated score which combined the PGS, we applied the following sensitivity analysis. For data that were available at a later point (later than the start date of the follow-up analysis; yellow in Figure S3B), we included them in the calculation of QRISK3 scores. We applied multiple imputation as described above and replaced the remaining missing data (red in Figure S3B) with imputed data. We then assessed the predictive accuracy of the new QRISK3 score and the integrated score. Results of the sensitivity analysis were in Supplementary Data 13.

Heritability estimation

We used unrelated individuals to estimate SNP heritability using GCTA-GREML. We excluded one sample in each pair of 3rd-degree relatives (kinship coefficient >0.0442 calculated using KING v2.2.4[1]). Age, sex, and first 10 genetic PCs were added as covariates. We first used SNPs with INFO >0.9 and MAF >0.01 to construct the GRM and calculate SNP heritability in each cohort separately. There are 5,122,196 and 2,965,005 SNPs available in eMERGE and G&H, separately. We also calculated SNP heritability using the intersection of these SNP sets in both cohorts (N=2,228,506), and we observed a similar trend with slightly smaller differences between the two cohorts (Supplementary Data 7).

Trans-ancestry genetic correlations

We calculated the trans-ancestry genetic correlations between G&H and UKBB European-ancestry individuals using Popcorn. The genetic correlation indicates the correlation of causal-variant effect sizes across the genome at SNPs common to both populations. Variant LD scores were estimated for ancestry-matched 1000 Genomes v3 data for each study combination (i.e. South Asian-European ancestry). The estimation of LD scores failed for

chromosome 6 for some groups, so we left out the major histocompatibility complex (MHC) region (positions 28,477,797 to 33,448,354) from chromosome 6 from all comparisons. Variants with INFO score <0.9 or MAF <0.01 were excluded.

Trans-ancestry colocalisation

We used the TAColoc [15] to perform the trans-ancestry colocalisation analysis. This method adopts the joint likelihood mapping (JLIM) statistic developed by Chun and colleagues [16] that estimates the posterior probabilities for colocalisation between GWAS signals and compares them to probabilities of distinct causal variants while explicitly accounting for LD structure. For this, LD scores were estimated using a subset of samples from the 1000 Genomes Project v3 that had matching ancestry to all Europeans for UK Biobank. For G&H we used raw genotype data and LD was estimated directly for these samples. JLIM assumes only one causal variant within a region in each study. We therefore used small windows of 50Kb for each known locus to minimise the risk of interference from additional association signals. Distinct causal variants were defined by separation in LD space by r^2 ≥0.8 from each other. We excluded loci where the overlap between UKBB and G&H was <10 SNPs and the proportion of well-imputed SNPs overlapping between cohorts (SNP coverage) was <10%; this left no loci to consider for CAD, SBP and DBP. We used a significance threshold of $p < 0.05$ to determine evidence of sharing. LocusZoom (http://locuszoom.org/) was to create regional association plots.

Comparison of performances of polygenic scores between ancestries

In this study, we compared the performances of PGSs in BPB people from the G&H cohort and EUR people from the eMERGE cohort. We calculated the PGSs in the two cohorts using

the two approaches described below. We reported the results using the PGS Catalog scores in the main text, because using previously published scores developed in external cohorts is less likely to have the issue of overfitting. We also compared the two approaches in Figure S9.

Previously developed scores from the PGS Catalog: we downloaded previously published PGSs from the PGS Catalog. These scores were developed mostly in European-ancestry populations. We restricted to 7,353,388 bi-allelic SNPs that had INFO ≥0.3 and MAF ≥0.1% in both eMERGE and G&H for scoring. There are often multiple PGSs available for the same trait, and we used the one with the highest accuracy in G&H and eMERGE, respectively. The PGS ID and the reference for the best score per trait in each of the two cohorts are in Supplementary Data 5.

Clumping and p-value thresholding (C+T) PGSs: we also calculated the PGSs based on the largest available GWAS summary data obtained in primarily European-ancestry populations (Supplementary Data 12). No GWAS have been conducted in large South Asian ancestry populations, thus using a much more powerful European-ancestry GWAS would lead to more predictive PGS in G&H. We used the clumping and p-value thresholding method (C+T) to calculate PGSs, which were optimised in G&H and eMERGE separately. We used LD estimated using European ancestry samples (N=503) from the 1000 Genomes project for clumping using PRSice2 v2.2.11 [17]. We calculated multiple scores using combinations of various LD r^2 thresholds (0.1, 0.2, 0.5, 0.8) and p-value thresholds (5×10^{-8}, 1×10^{-7}, 5×10^{-7}, 1×10^{-6}, 5×10^{-6}, 1×10^{-5}, 5×10^{-5}, 1×10^{-4}, 5×10^{-4}, 0.001, 0.005, 0.01, 0.02, 0.05, 0.08, 0.1, 0.2, 0.3, 0.4, 0.5, 0.8, 1) for each trait. We would suffer from overfitting if we report the performance of the PGS that is optimised in the same cohort. To avoid overfitting, we applied 10-fold cross validation and repeated it 100 times [18,19]. For CAD, the 10 folds had the same case-control ratio. For each left-out fold, we calculated the prediction accuracy for PGS constructed using

parameters that were selected in the other nine folds. We then calculated the average across the 10 folds and 100 repeats and reported the average performances and the parameters of the most frequently selected PGS in Supplementary Data 12. The 95% confidence intervals were also calculated from the 10 folds and 100 repeats.

Meta-PGSs combining ancestry-specific GWAS

In G&H, we further assessed the performance of PGSs that incorporated GWAS summary data from the target non-European populations (South Asian ancestry in our case) to improve cross-ancestry prediction. We downloaded GWAS summary data that were generated in South Asian samples of the UKBB from the Pan-UK Biobank website (https://pan.ukbb.broadinstitute.org) to construct South Asian-specific PGS.

Meta-PGS by Marquez-Luna *et al.* [20]: We constructed scores (PGS_{SAS}) using the C+T method described above and using South Asian samples from the 1000 Genomes project for the LD reference. We evenly split the G&H cohort into a tuning set and a validation set. We performed a linear/logistic regression in the tuning set and estimated the weights for PGS_{EUR} (which were downloaded from the PGS Catalog, described in Methods) and PGS_{SAS}. We then calculated the meta-PGS by linearly combining the two scores and tested the performance in the validation set. We repeated it for 100 times and calculated the average accuracy across the 100 random splits and estimated the 95% confidence interval.

PRS-CSx: We applied PRS-CSx, an extension of a Bayesian method (PRS-CS), which enabled more accurate estimation of effect sizes by leveraging population-specific LD and shared genetic information between populations through joint modelling of multiple GWAS summary data [21]. We used publicly available GWASs in Supplementary Data 4 to construct the European-specific PGS. LD reference panels constructed using the 1000 Genomes

Project were downloaded from https://github.com/getian107/PRScsx. Following Ruan *et al.* [21], we restricted to common variants available in HapMap3. Similarly, we linearly combined the PRS-CSx-auto scores derived from European ancestry GWASs (PGS_{EUR}) and from pan UKBB South Asian samples (PGS_{SAS}), for which the weights were estimated in the tuning set, and accessed the performance of the combined score in the validation set. The performance was calculated as the average across 100 random splits and the 95% confidence interval was estimated from them.

Supplementary Figures

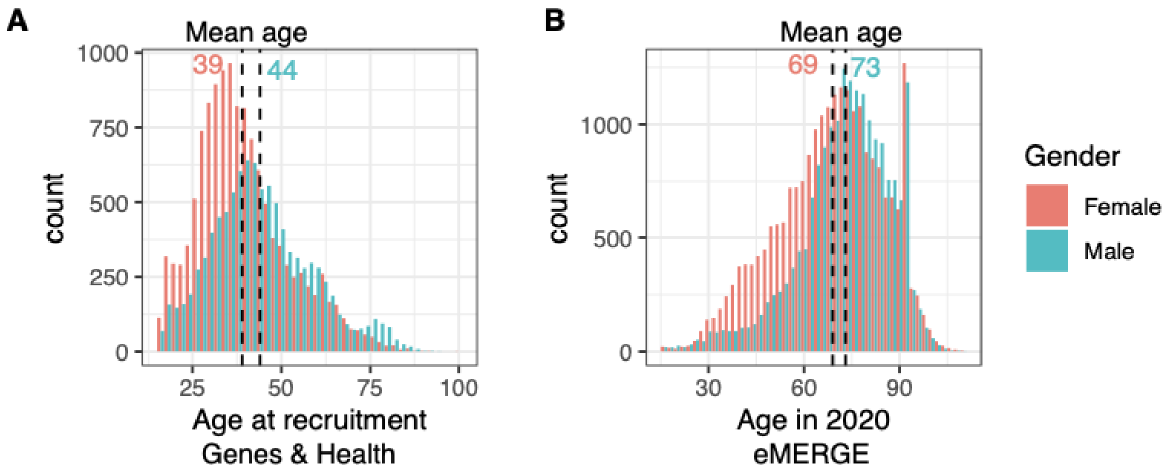

Supplementary Figure 1. Age distributions of G&H (A) and eMERGE (B). Red indicates female participants and blue indicates male participants. Vertical dashed lines indicate the average age. In G&H, 56.5% of the 22,490 individuals with electronic health record data are female, with the mean age 39.4 (standard deviation, SD: 13.1) years old for women and 44.3 (SD: 14.3) for men. In eMERGE, 54.5% of the 42,802 individuals are female, with the mean age 69.1 (SD: 16.7) and 73.1 (SD: 14.7) for women and men, respectively.

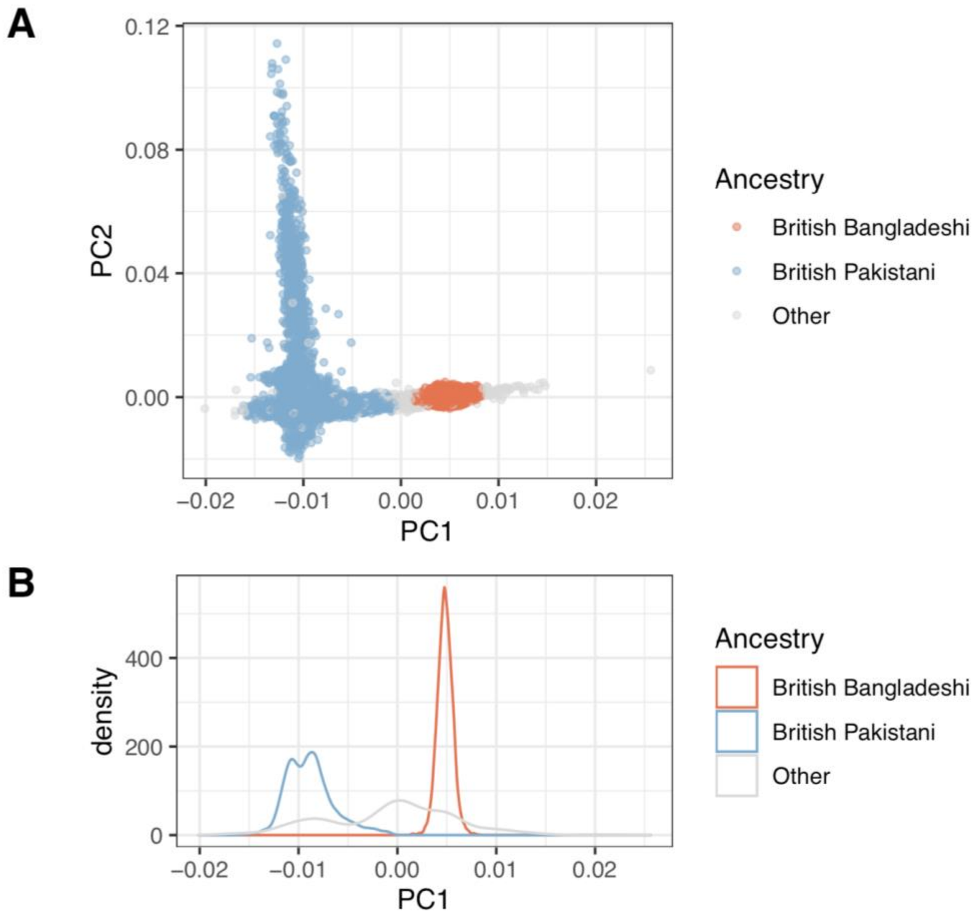

Supplementary Figure 2. Principal component analysis (PCA) of 28,022 genotyped individuals from the Genes & Health (G&H) cohort. A. PC1 and PC2 for all samples. Red indicates self-declared British Bangladeshi samples (N=17,721) and blue represents self-declared British Pakistani samples (N=9,694). We excluded samples who self-reported as coming from other ethnic groups (“Other”) or who did not report ethnicity information, as well as genetically-inferred outliers (those with PC1 further than +/- 3 standard deviations from the mean of PC1 for the individuals who self-reported as coming from that group). These samples are in grey (N=607). **B**. Density plot for PC1, which clearly differentiates self-declared British Bangladeshis from self-declared British Pakistanis.

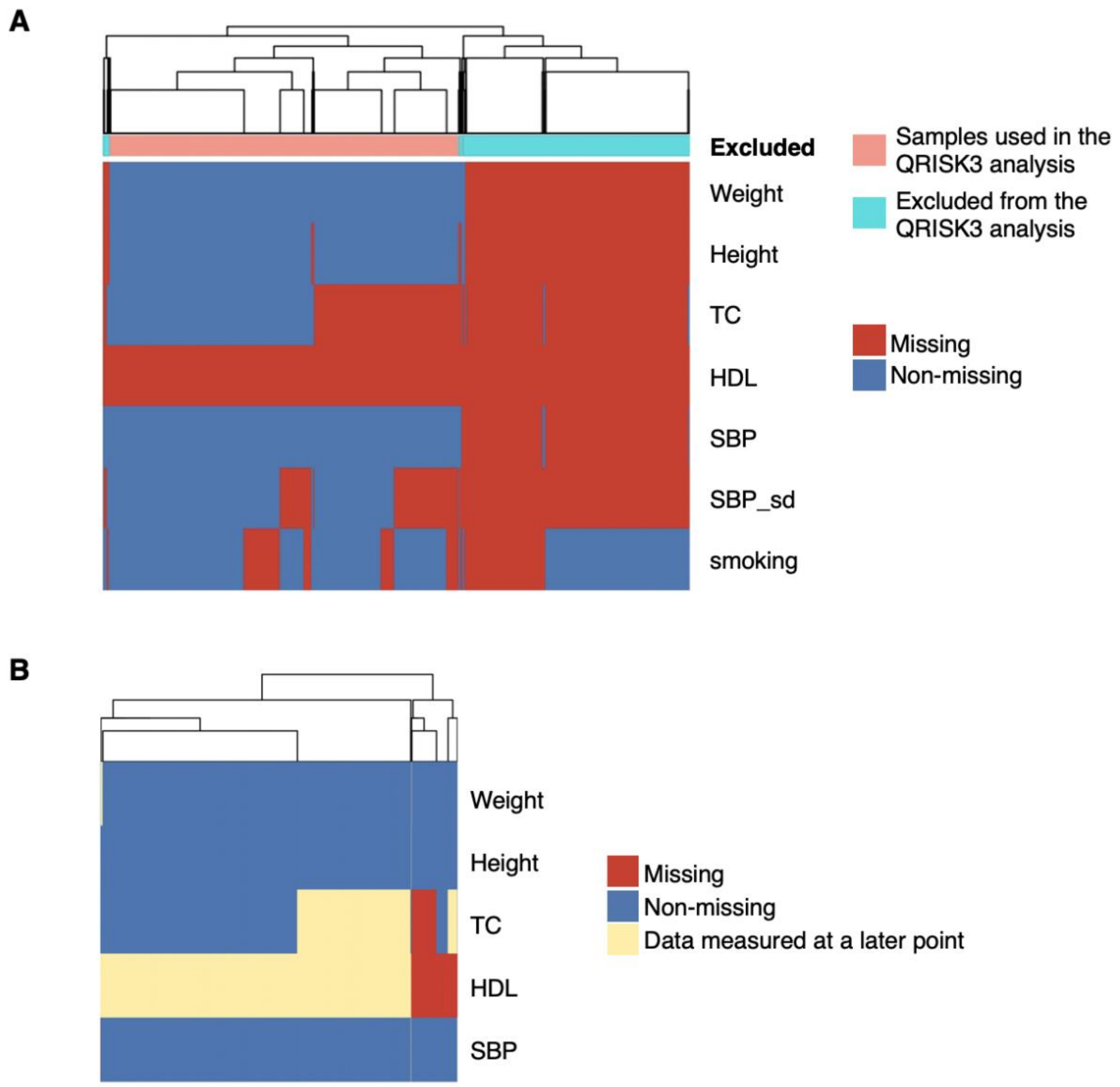

Supplementary Figure 3. Pattern of missingness for continuous variables in the QRISK3 algorithm. (**A**) Columns represent all individuals who were aged 25-84 years old in 2010 (N=15,890). Prevalent cases who developed coronary artery disease earlier than 2010 were excluded from the QRISK3 analysis. Rows represent variables. Missing data are in red. On the top of the heatmap, green indicates individuals that were excluded from the QRISK3 analysis because of high missingness. (**B**) Columns represent individuals that were included in the QRISK3 analysis (N=9,477). Data that were measured earlier than 1st Jan, 2010 are in blue. Yellow indicates data that were measured at a later point. Data in blue and yellow were used in the sensitivity analysis (Supplementary Methods), and we replaced missing data (red) with imputed data. TC: total cholesterol; HDL: high-density lipoprotein cholesterol; SBP: systolic blood pressure; SBP_sd: standard deviation of SBP measurements within 2 years.

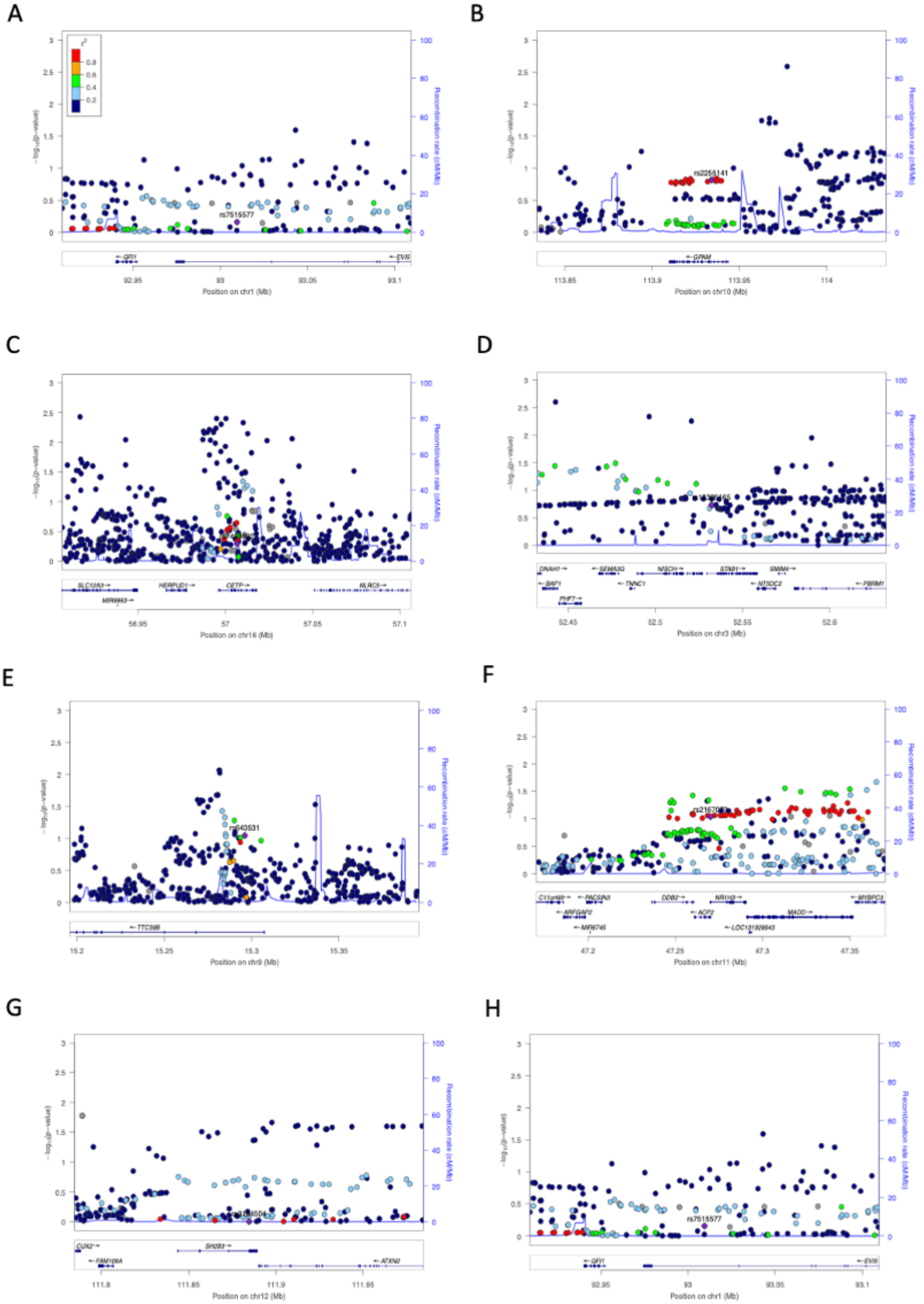
A
B
C
D
E
F
G
H
Position on chr1 (Mb)
Position on chr10 (Mb)
Position on chr16 (Mb)
Position on chr3 (Mb)
Position on chr9 (Mb)
Position on chr11 (Mb)
Position on chr12 (Mb)
Position on chr1 (Mb)
Recombination rate (cM/Mb)
rs7515577
rs2255141
rs643531
rs7515577

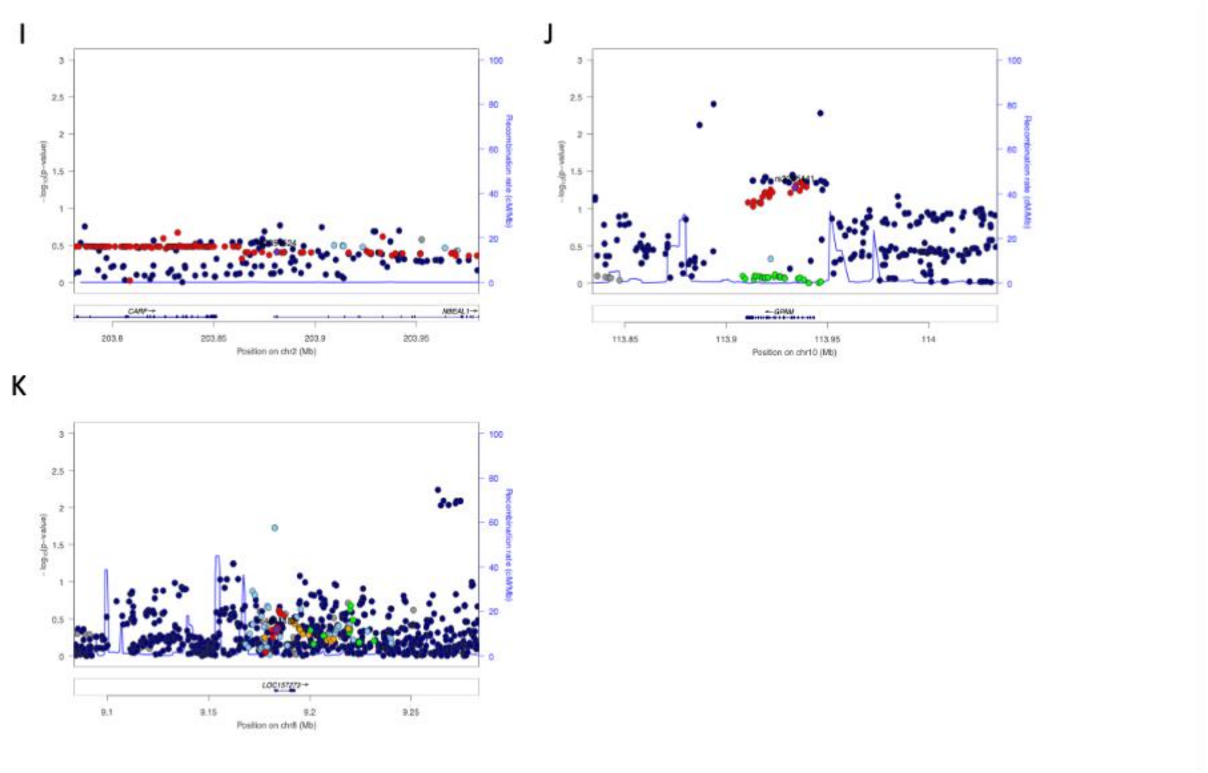

Supplementary Figure 4. Regional association plots for non-transferable loci in G&H (Credible set p > 0.05 and no variant within 50kb of locus with p < 1×10^{-3}). **A.** LDL-C, *EVI5* locus (rs7515577). **B.** LDL-C, *GPAM* locus (rs2255141). **C.** LDL-C, *CETP* locus (rs7499892). **D**. HDL-C STAB1 locus (rs13326165). **E.** HDL-C, *TTC39B* locus (rs643531). **F.** HDL-C, *ACP2* locus (rs2167079). **G.**HDL-C, *SH2B3* locus (rs3184504). **H.** Total Cholesterol, *EVI5* locus (rs7515577). **I.** Total Cholesterol, *NBEAL1* locus (rs2351524). **J.** Total Cholesterol, *GPAM* locus (rs2255141). **K.** Triglycerides, *NECAP2* locus (rs4841132). Colour of the points corresponds to the strength of linkage disequilibrium (r^2) of each potential causal variant (in brackets) identified in EUR ancestry labelled and coloured in purple.

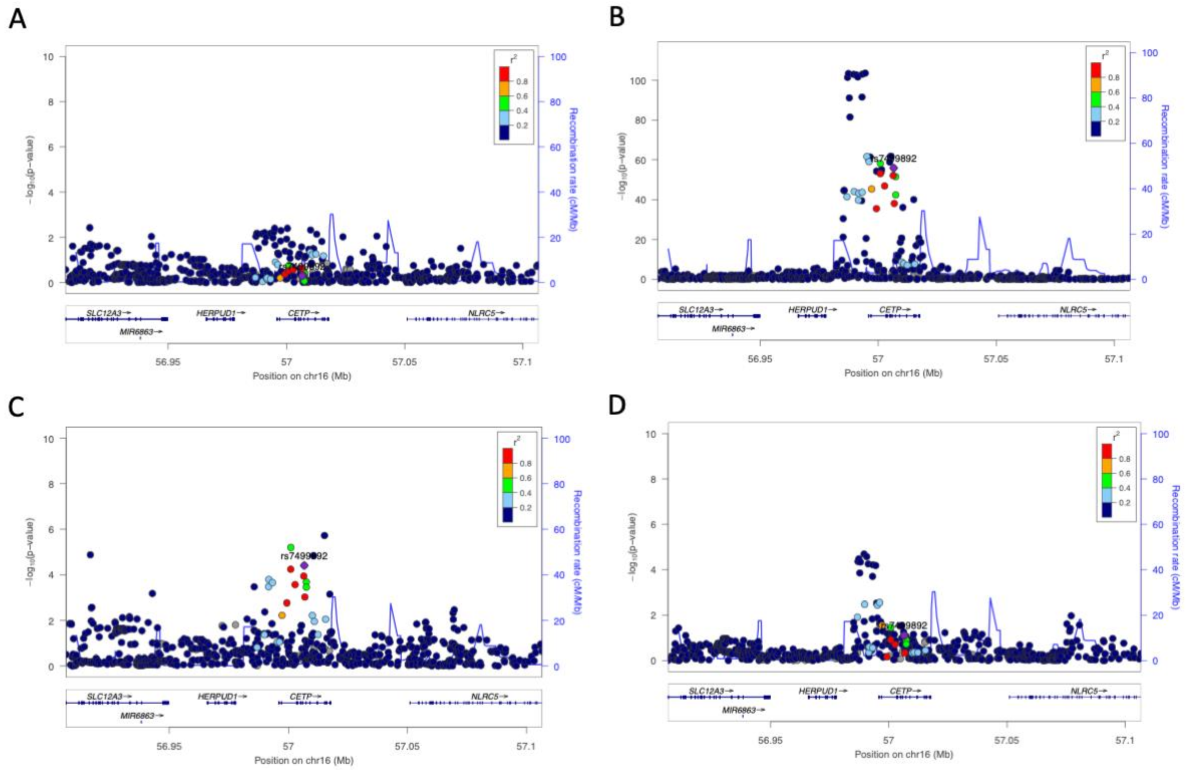

Supplementary Figure 5. Regional association plots for *CETP* locus across lipid traits in G&H. **A.** LDL-C. **B.** HDL-C. **C.** Total Cholesterol. **D.** Triglycerides. Colour of the points corresponds to the strength of linkage disequilibrium (r^2) of potential causal variant (rs7499892) identified in European ancestry, labelled and coloured in purple.

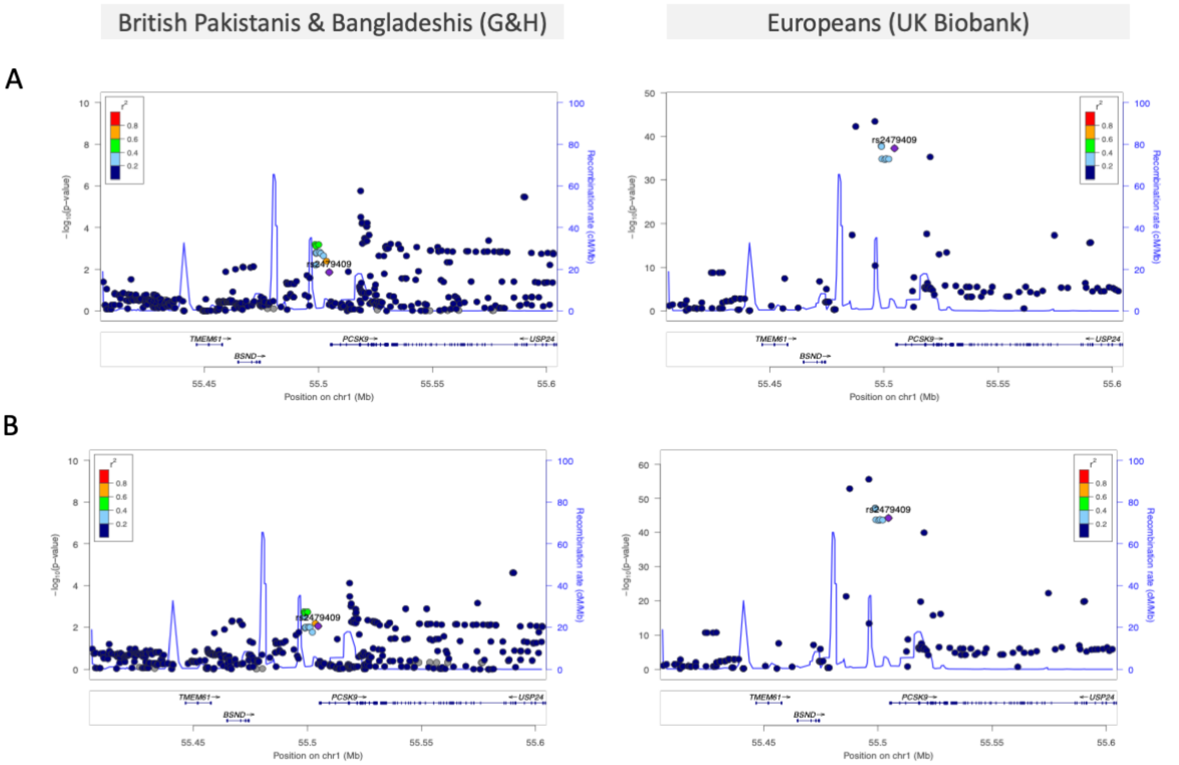

Supplementary Figure 6. Regional association plots for major lipid locus, *PCSK9* with unshared causal variant rs2479409 (p_{JLIM}>0.05) between British Pakistanis and Bangladeshis (G&H) and Europeans (UK Biobank). A. LDL-C. **B.** Total Cholesterol. Colour of the points corresponds to the strength of linkage disequilibrium (r^2) of each potential causal variant (in brackets) identified in European ancestry, labelled and coloured in purple.

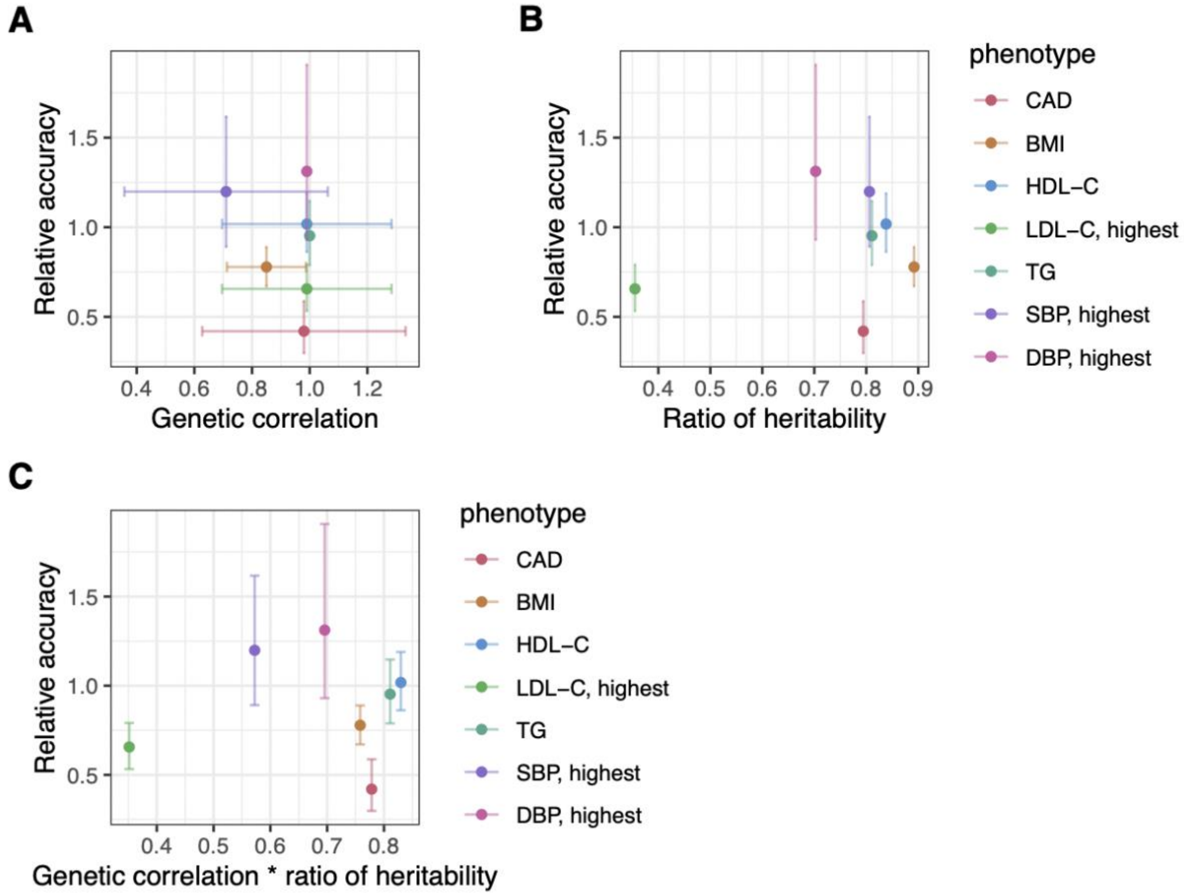

Supplementary Figure 7. Relationship between the relative accuracy of polygenic scores (PGSs) and trans-ancestry genetic correlation and heritability estimates. We used PGSs from the PGS Catalog. We show the relationship between relative accuracies of PGSs (i.e. the ratio of incremental AUC for coronary artery disease (CAD) or incremental R^2 for risk factors estimated in G&H to that in eMERGE) on the y-axes versus the trans-ancestry genetic correlation (**A**), ratio of the heritability estimates in G&H over UKB (**B**), and the product of the previous two terms (**C**). None of the correlations is significant. Error bars show 95% confidence intervals (CIs) on both axes. The 95% CIs for relative accuracy on y-axes were estimated from bootstrap resampling (n=1,000 times) and that for genetic correlation on x-axis in (**A**) were estimated using the standard error. For G&H, heritability and accuracy of PGS were estimated in n=17,348 (996 cases) unrelated samples for CAD, n=13,926 for body-mass index (BMI), n=11,316 for high-density lipoprotein cholesterol (HDL-C), n=12,856 for low-density lipoprotein cholesterol (LDL-C), n=11,125 for triglycerides (TG), and n=15,908 for both systolic blood pressure (SBP) and diastolic blood pressure (DBP). For eMERGE, heritability and accuracy of PGS were estimated in n=32,816 (6,815) unrelated samples for CAD, n=37,160 for BMI, n=16,049 for HDL-C, n=15,856 for LDL-C, n=14,384 for TG, and n=11,864 for both SBP and DBP.

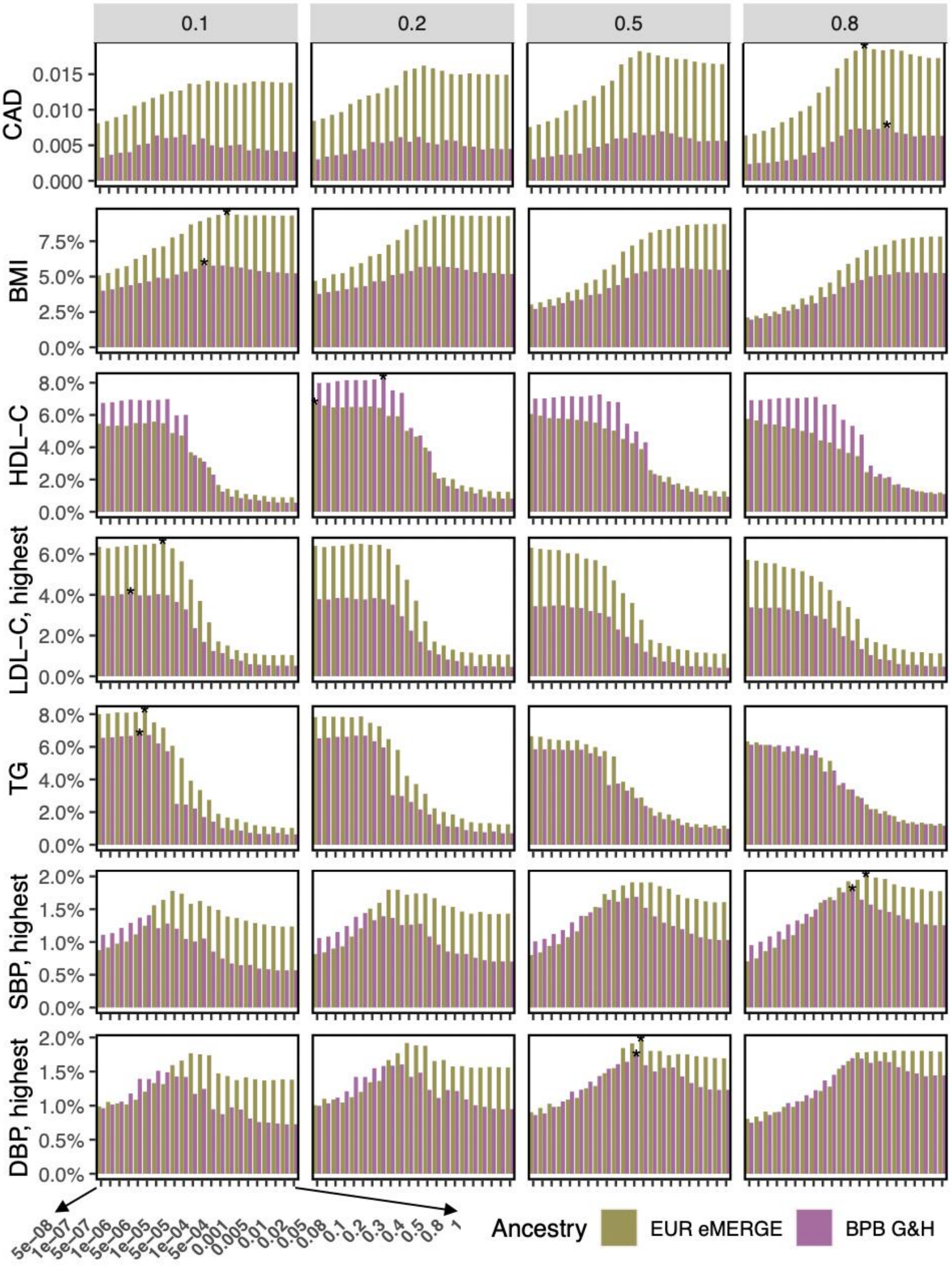

Supplementary Figure 8. Predictive performance of polygenic scores (PGSs) across LD r^2 and p-value thresholds. Incremental AUC is shown for coronary artery disease (CAD) and incremental R^2 is shown for the continuous risk factors. Purple indicates the performance of the PGS selected using 10-fold cross validation (Supplementary Methods) in all G&H samples and green in eMERGE. This is for the purpose of visualisation and the results reported in the manuscript are from cross validation. Bars represent PGSs constructed using combinations of various p-values (on x-axes; unadjusted, two-sided p-

values were from publicly available GWAS datasets) and LD clumping r^2 thresholds (in the four columns). PGSs are sorted so that those on the right contain more SNPs. Asterisks indicate the reported PGSs with the highest accuracy per trait. For G&H, the accuracy of PGS was estimated in n=17,348 (996 cases) unrelated samples for CAD, n=13,926 for body-mass index (BMI), n=11,316 for high-density lipoprotein cholesterol (HDL-C), n=12,856 for low-density lipoprotein cholesterol (LDL-C), n=11,125 for triglycerides (TG), and n=15,908 for both systolic blood pressure (SBP) and diastolic blood pressure (DBP). For eMERGE, the accuracy of PGS was estimated in n=32,816 (6,815) unrelated samples for CAD, n=37,160 for BMI, n=16,049 for HDL-C, n=15,856 for LDL-C, n=14,384 for TG, and n=11,864 for both SBP and DBP.

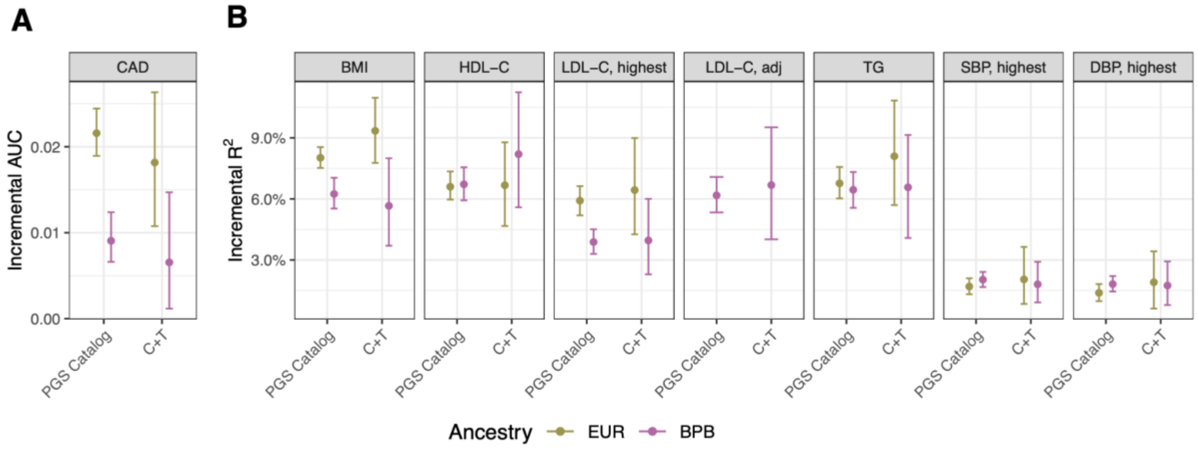

Supplementary Figure 9. Comparing polygenic scores (PGSs) from the PGS Catalog and scores calculated using the Clumping and P-value thresholding (C+T) method. Predictive accuracy of PGSs for cardiometabolic traits in British Pakistani and Bangladeshi (BPB) individuals from G&H (purple) and European-ancestry (EUR) individuals from eMERGE (green). Incremental AUC (**A**) was calculated for coronary artery disease (CAD), and incremental R^2 (**B**) was calculated for its continuous risk factors. Previously published scores downloaded from the PGS Catalog were developed in mostly European-ancestry populations (Supplementary Data 5). We also calculated PGSs based on publicly available GWAS summary statistics (Supplementary Data 12) using the C+T method. The C+T PGSs were tuned in G&H and eMERGE, respectively (Supplementary Methods). For C+T scores, we repeated 10-fold cross validation 100 times, and calculated the average accuracy. Error bars indicate 95% confidence intervals estimated by bootstrap resampling of samples for PGS Catalog scores and repeating 10-fold cross validation for C+T scores. The highest measurements for low-density lipoprotein cholesterol (LDL-C), systolic blood pressure (SBP), and diastolic blood pressure (DBP) are compared between eMERGE and G&H, and statin-adjusted LDL-C data are also shown for G&H. For G&H, the accuracy of PGS was estimated in n=17,348 (996 cases) unrelated samples for CAD, n=13,926 for body-mass index (BMI), n=11,316 for HDL-C, n=12,856 for the highest LDL-C measurement, n=10,939 for statin-adjusted LDL-C, n=11,125 for triglycerides (TG), and n=15,908 for both SBP and DBP. For eMERGE, the accuracy of PGS was estimated in n=32,816 (6,815) unrelated

samples for CAD, n=37,160 for BMI, n=16,049 for HDL-C, n=15,856 for LDL-C, n=14,384 for TG, and n=11,864 for both SBP and DBP.

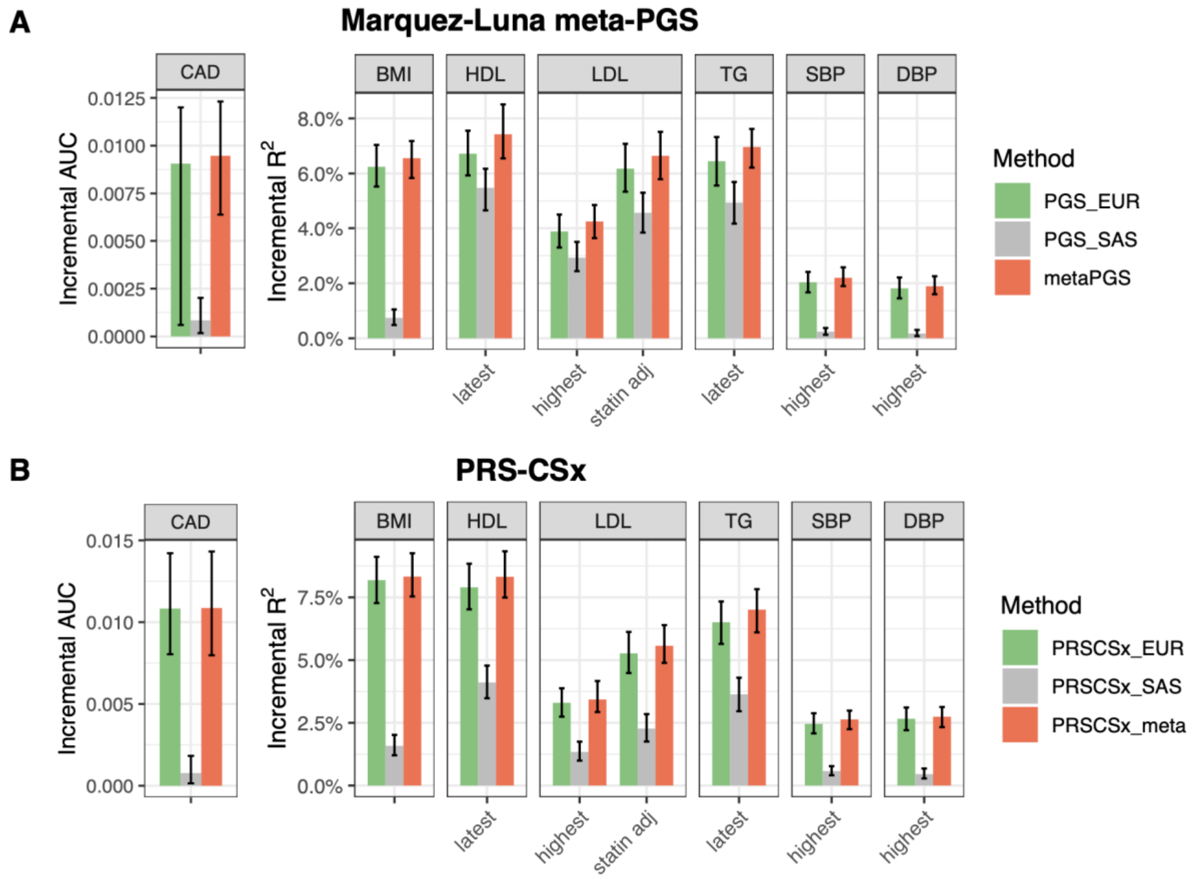

Supplementary Figure 10. Predictive performance of polygenic scores (PGSs) that incorporate GWAS data from ancestry-matched samples. PGSs were constructed using the Marquez-Luna method (**A**) and PRS-CSx (**B**), incorporating GWAS data from the UK Biobank South Asian-ancestry (SAS) individuals (Supplementary Methods). Incremental AUC was calculated for CAD, and incremental R^2 was calculated for its continuous risk factors. Error bars indicate 95% confidence intervals which were estimated from bootstrap resampling (N=1,000). Colours indicate PGSs that are constructed using different GWAS training data (green: GWAS statistics from EUR studies; grey: GWAS statistics from SAS samples from UKBB; red: the final PGS derived from linearly combining the previous two PGSs). (**A**) Improvement in accuracy comparing the meta-PGSs (red) with the EUR PGS (green) is 4.5% for CAD, 5.0% for BMI, 10.5% for HDL-C, 9.7% for the highest LDL-C, 7.6% for the statin-adjusted LDL-C, 8.0% for TG, 8.0% for SBP, and 4.2% for DBP. (**B**) Improvement in accuracy comparing the final PRS-CSx score (red) with the EUR score (green) is 0.3% for CAD, 1.7% for BMI, 5.3% for HDL-C, 4.0% for the highest LDL-C, 5.7% for the statin-adjusted LDL-C, 7.7% for TG, 7.2% for SBP, and 2.3% for DBP. The accuracy of PGS was estimated in n=17,348 (996 cases) unrelated samples from G&H for CAD, n=13,926 for BMI, n=11,316 for HDL-C, n=12,856 for the highest LDL-C measurement, n=10,939 for statin-adjusted LDL-C, n=11,125 for triglycerides (TG), and n=15,908 for both SBP and DBP.

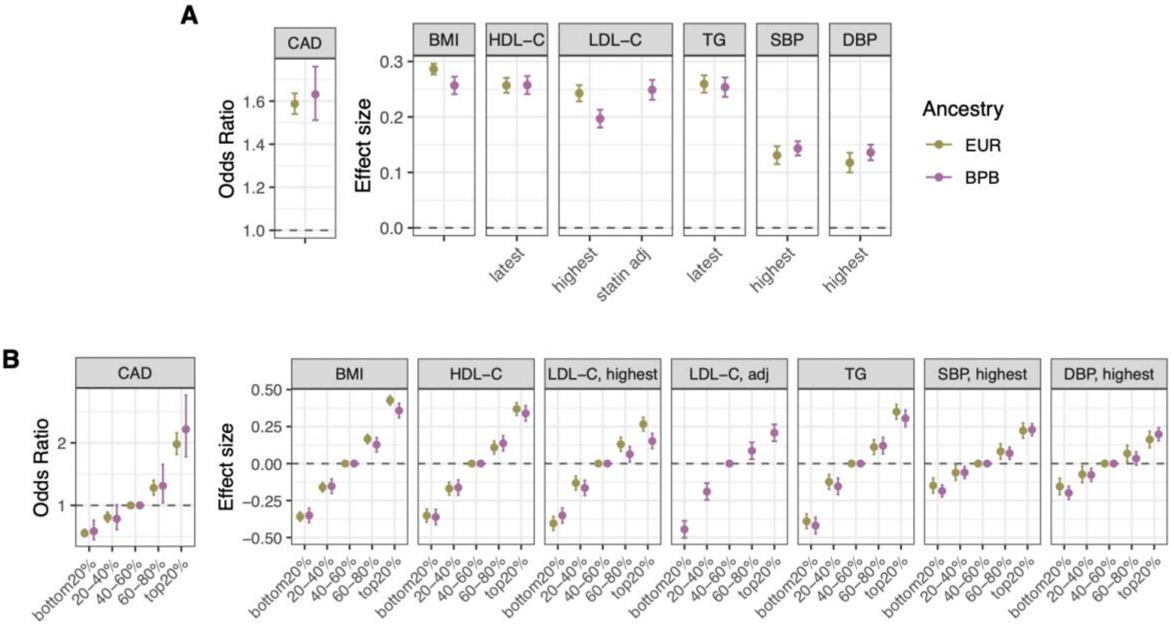

Supplementary Figure 11. Effect sizes of polygenic scores (PGSs) from the PGS Catalog. A. The odds ratio per standard deviation (SD) of PGS is shown for coronary artery disease (CAD) on the left panel, and the differences in phenotypic SD per SD of PGS are shown for quantitative traits on the right panel. **B**. The odds ratio for CAD comparing the four quintiles to the middle quintile (40–60%) is shown on the left panel. Quintiles are determined in controls. The differences in phenotypic SD compared to the reference quintile are shown on the right panel. Error bars show 95% confidence intervals estimated using the standard error. For G&H, the effect sizes of PGS in both (**A**) and (**B**) were estimated in n=17,348 (996 cases) unrelated samples for CAD, n=13,926 for body-mass index (BMI), n=11,316 for high-density lipoprotein cholesterol (HDL-C), n=12,856 for the highest low-density lipoprotein cholesterol (LDL-C) measurement, n=10,939 for statin-adjusted LDL-C, n=11,125 for triglycerides (TG), and n=15,908 for both systolic blood pressure (SBP) and diastolic blood pressure (DBP). For eMERGE, the effect sizes of PGS were estimated in n=32,816 (6,815) unrelated samples for CAD, n=37,160 for BMI, n=16,049 for HDL-C, n=15,856 for LDL-C, n=14,384 for TG, and n=11,864 for both SBP and DBP.

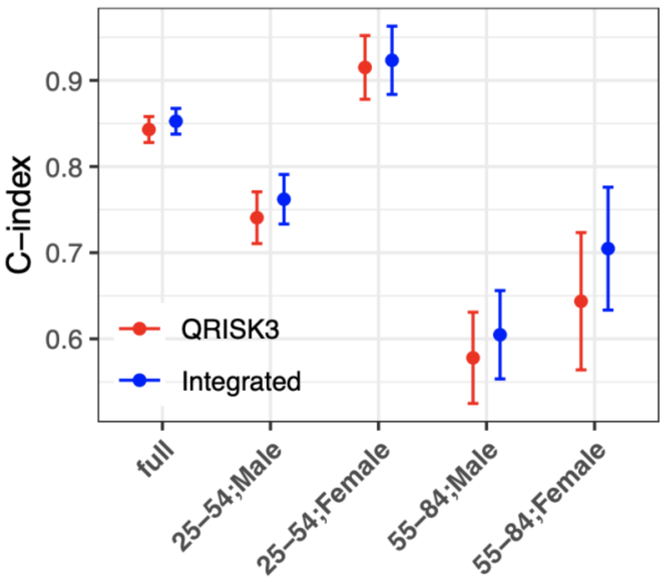

Supplementary Figure 12. Model discrimination for coronary artery disease (CAD) with addition of a polygenic score to QRISK3. Red indicates the concordance index (C-index) of QRISK3 and blue indicates the C-index of an integrated score that combines QRISK3 and a polygenic score for CAD. Estimates in all British Pakistani and Bangladeshi individuals (n=420 unrelated cases and 7,702 unrelated non-cases) from G&H as well as in age-by-sex subgroups (n=207 and 2,779 in males aged 25–54; n=51 and 4,187 in females aged 25–54; n=114 and 344 in males aged 55–84; n=48 and 392 in females aged 55–84) are shown. The error bars represent 95% confidence intervals estimated using the standard error.

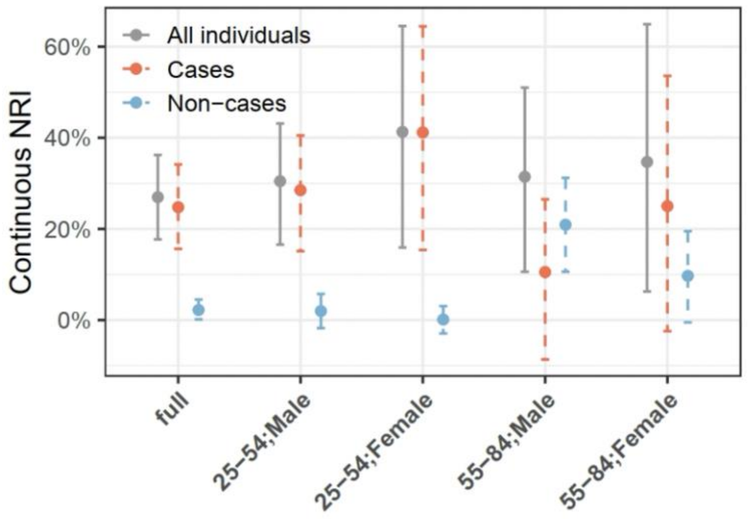

Supplementary Figure 13. Continuous net reclassification index (NRI) for the integrated score compared to QRISK3 in all samples and age-by-gender subgroups. Continuous NRI in cases (red) and non-cases (blue) are shown. The error bars indicate 95% confidence intervals estimated using the bootstrap method. Overall continuous NRI was estimated in n=420 unrelated cases and 7,702 unrelated non-cases from G&H. NRI in age-by-sex subgroups was estimated with the following sample sizes: n=207 unrelated cases

and 2,779 unrelated non-cases in males aged 25–54; n=51 and 4,187 in females aged 25–54; n=114 and 344 in males aged 55–84; n=48 and 392 in females aged 55–84.

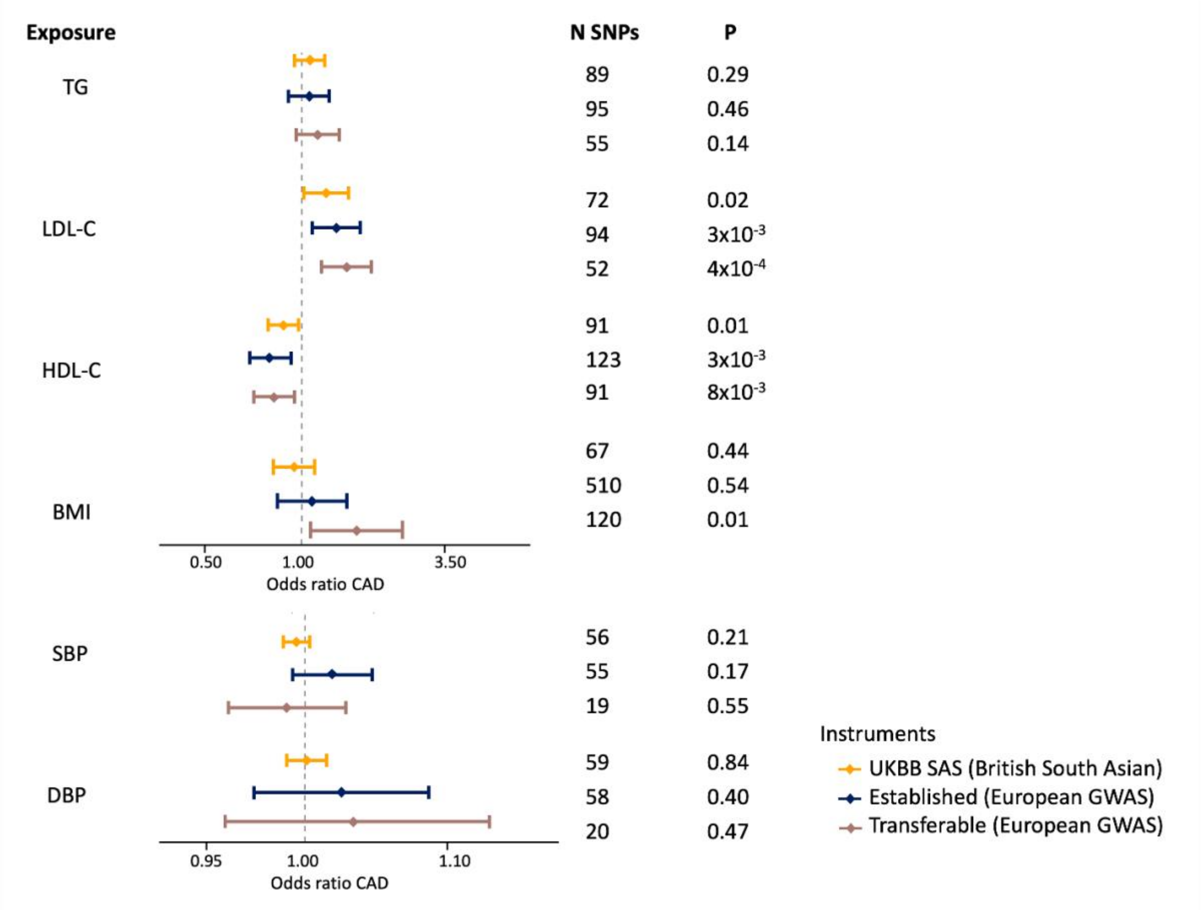

Supplementary Figure 14. Mendelian randomisation estimates of risk factors on coronary artery disease using different strategies for instrument selection. Two-sample Mendelian Randomisation (MR) using coronary artery disease risk (CAD) in G&H (n=22,008 samples; among them 1,110 were cases) as the outcome. Genetic instrumental variables for the risk factors were selected based on different strategies: loci associated at $p<1x10^{-5}$ (unadjusted, two-sided p-values were from publicly available GWAS datasets) in an ancestry-matched GWAS (UKBB SAS), all genome-wide significant loci from the largest EUR GWAS, and the subset of these loci that were transferable to SAS. Effect estimates are presented as odds ratios with 95% confidence intervals per standard deviation increase in the reported unit of the trait: triglycerides (TG), systolic blood pressure (SBP), low-density lipoprotein cholesterol (LDL-C), high-density lipoprotein cholesterol (HDL-C), diastolic blood pressure (DBP), body mass index (BMI). The p-value (P) and number of single nucleotide polymorphism instruments (N SNP) included in the MR analysis are shown for each exposure.

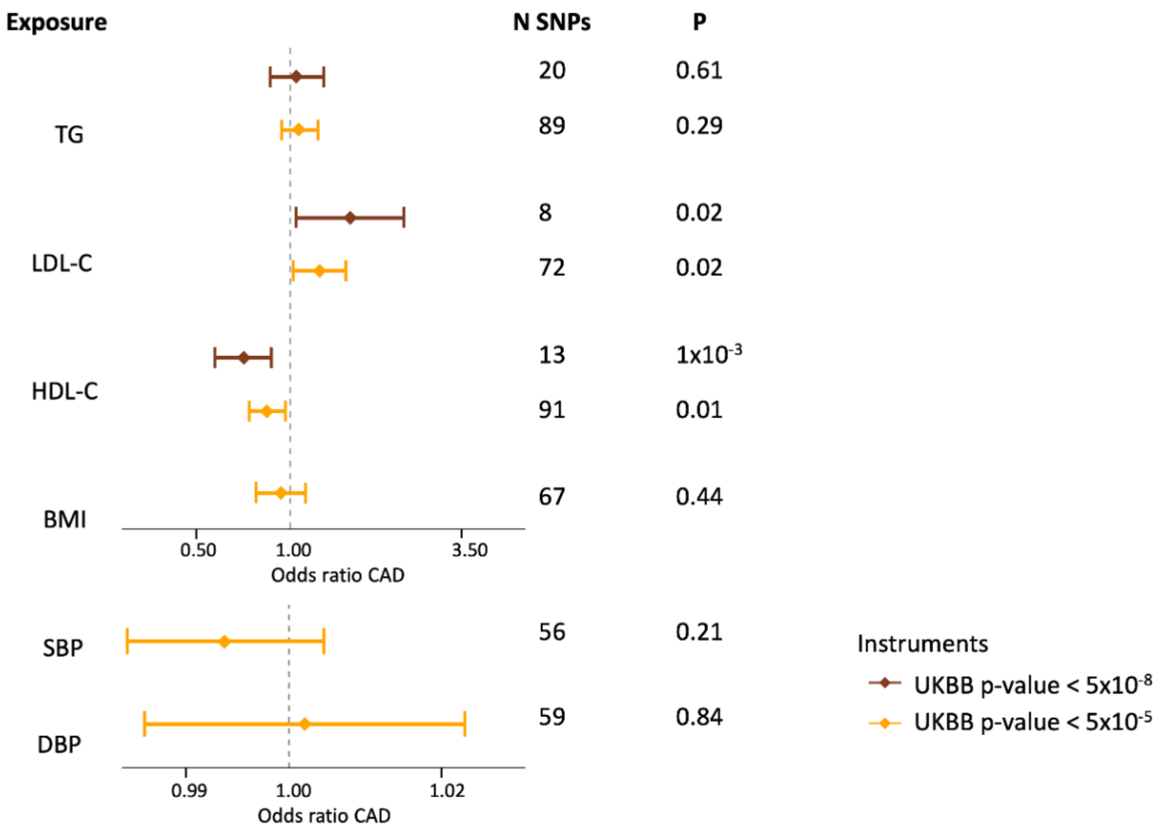

Supplementary Figure 15. Mendelian randomisation estimates of the causal effects of risk factors on coronary artery diseases (CAD) in G&H using loci from ancestry-matched discovery GWAS as instruments. GWAS for CAD was performed in n=22,008 (1,110 cases) samples from G&H. Association of risk factors with CAD was assessed for instruments selected from UKBB SAS at two p-value thresholds (unadjusted, two-sided p-values were from publicly available GWAS datasets): $p<5\times10^{-5}$ and $p<5\times10^{-8}$. Effect estimates are presented as odds ratios with 95% confidence intervals per standard deviation increase in the reported unit of the trait: triglycerides (TG), systolic blood pressure (SBP), low-density lipoprotein cholesterol (LDL-C), high-density lipoprotein cholesterol (HDL-C), diastolic blood pressure (DBP), body mass index (BMI). The p-value (P) and number of SNP instruments (N SNP) included in the MR analysis are shown for each exposure.

Genes & Health Research Team

List of current members (in alphabetical order by surname): Shaheen Akhtar, Mohammad Anwar, Elena Arciero, Samina Ashraf, Gerome Breen, Raymond Chung, Charles J Curtis, Maharun Chowdhury, Grainne Colligan, Panos Deloukas, Ceri Durham, Sarah Finer, Chris Griffiths, Qin Qin Huang, Matt Hurles, Karen A Hunt, Shapna Hussain, Kamrul Islam, Ahsan Khan, Amara Khan, Cath Lavery, Sang Hyuck Lee, Robin Lerner, Daniel MacArthur, Bev MacLaughlin, Hilary Martin, Dan Mason, Shefa Miah, Bill Newman, Nishat Safa, Farah Tahmasebi, Richard C Trembath, Bhavi Trivedi, David A van Heel, John Wright

Supplementary References

1. Manichaikul, A. *et al.* Robust relationship inference in genome-wide association studies. *Bioinformatics* **26**, 2867–2873 (2010).
2. Patterson, N., Price, A. L. & Reich, D. Population structure and eigenanalysis. *PLoS Genet.* **2**, e190 (2006).
3. McInnes, L., Healy, J. & Melville, J. UMAP: Uniform Manifold Approximation and Projection for Dimension Reduction. *arXiv [stat.ML]* (2018).
4. Discovery Data Service. https://wiki.discoverydataservice.org/index.php?title=Welcome_to_the_Discovery_Data_Service_knowledge_base.
5. NHS UK Read Codes · TRUD. https://isd.digital.nhs.uk/trud3/user/guest/group/0/pack/9.
6. Finer, S. *et al.* Cohort Profile: East London Genes & Health (ELGH), a community-based population genomics and health study in British Bangladeshi and British Pakistani people. *Int. J. Epidemiol.* **49**, 20–21i (2020).
7. Nelson, C. P. *et al.* Association analyses based on false discovery rate implicate new loci for coronary artery disease. *Nat. Genet.* **49**, 1385–1391 (2017).
8. Liu, D. J. *et al.* Exome-wide association study of plasma lipids in >300,000 individuals. *Nat. Genet.* **49**, 1758–1766 (2017).

9. Evangelou, E. *et al.* Genetic analysis of over 1 million people identifies 535 new loci associated with blood pressure traits. *Nat. Genet.* **50**, 1412–1425 (2018).
10. Sham, P. C. & Purcell, S. M. Statistical power and significance testing in large-scale genetic studies. *Nat. Rev. Genet.* **15**, 335–346 (2014).
11. Matti Pirinen, University of Helsinki. GWAS 3: Statistical power. https://www.mv.helsinki.fi/home/mjxpirin/GWAS_course/material/GWAS3.html.
12. Riveros-Mckay, F. *et al.* An Integrated Polygenic Tool Substantially Enhances Coronary Artery Disease Prediction. *Circ Genom Precis Med* (2021) doi:10.1161/CIRCGEN.120.003304.
13. Yousaf, S. & Bonsall, A. UK Townsend Deprivation Scores from 2011 census data. *Colchester, UK: UK Data Service* (2017).
14. Wang, M. *et al.* Validation of a Genome-Wide Polygenic Score for Coronary Artery Disease in South Asians. *J. Am. Coll. Cardiol.* **76**, 703–714 (2020).
15. Kuchenbaecker, K. *et al.* The transferability of lipid loci across African, Asian and European cohorts. *Nat. Commun.* **10**, 4330 (2019).
16. Chun, S. *et al.* Limited statistical evidence for shared genetic effects of eQTLs and autoimmune-disease-associated loci in three major immune-cell types. *Nat. Genet.* **49**, 600–605 (2017).
17. Choi, S. W. & O'Reilly, P. F. PRSice-2: Polygenic Risk Score software for biobank-scale data. *Gigascience* **8**, (2019).
18. Krapohl, E. *et al.* Multi-polygenic score approach to trait prediction. *Mol. Psychiatry* **23**, 1368–1374 (2018).
19. Kim, J.-H. Estimating classification error rate: Repeated cross-validation, repeated hold-out and bootstrap. *Comput. Stat. Data Anal.* **53**, 3735–3745 (2009).
20. Márquez-Luna, C., Loh, P.-R., South Asian Type 2 Diabetes (SAT2D) Consortium, SIGMA Type 2 Diabetes Consortium & Price, A. L. Multiethnic polygenic risk scores improve risk prediction in diverse populations. *Genet. Epidemiol.* **41**, 811–823 (2017).
21. Ruan, Y. *et al.* Improving polygenic prediction in ancestrally diverse populations. *bioRxiv* (2021) doi:10.1101/2020.12.27.20248738.
