# Supplementary material for: Transferability of genetic loci and polygenic scores for cardiometabolic traits in British Pakistani and Bangladeshi individuals
Source: Nat Commun. 2022 Aug 9;13:4664. doi: 10.1038/s41467-022-32095-5 (PMC9363492; doi:10.1038/s41467-022-32095-5)
Supplement: Supplementary file 3 — Description of Additional Supplementary Files [file 41467_2022_32095_MOESM3_ESM.pdf]

Description of Additional Supplementary Files

File Name: Supplementary Data 1
Description: Definition of coronary artery disease (CAD). In G&H, diagnosis codes from hospitals were in the ICD10 system. READ codes from the primary care system were converted to SNOMED CT codes. Procedure codes from hospitals were in the OPCS system. In eMERGE, diagnosis codes were in the ICD10 or ICD9 systems. "%" indicates that we included all codes linked to that parent code.

File Name: Supplementary Data 2
Description: Sample sizes (and number of cases for CAD) of European-ancestry individuals from eMERGE and British Pakistani and Bangladeshi individuals from G&H. PGS analyses were restricted to unrelated samples.

File Name: Supplementary Data 3
Description: Definitions of variables in the QRISK3 algorithm. In G&H, diagnosis codes from hospitals were in the ICD10 system. READ codes from the primary care system were converted to SNOMED CT codes. Procedure codes from hospitals were in the OPCS system. "%" indicates that we included all codes linked to that parent code.

File Name: Supplementary Data 4
Description: Publicly available GWAS summary datasets used in each analysis. For SBP and DBP, summary statistics for proxy SNPs (SNPs that are in credible sets) were generated in UK Biobank EUR samples downloaded from the GWAS Atlas (https://atlas.ctglab.nl/traitDB/3380 and https://atlas.ctglab.nl/traitDB/3379), because the full summary data from Evangelou et al. are not publicly available. Evangelou et al. used raw blood pressure data and our power calculation requires effect sizes estimated using standardised phenotype data, so we used effect sizes from UK Biobank GWAS. We used UK Biobank GWAS data to construct PGS for SBP and DBP, and for the remaining traits we used the same GWAS as was used for determining the 'established loci' for assessing their transferability.

File Name: Supplementary Data 5
Description: Performance of polygenic scores (PGSs) from the PGS Catalog in G&H versus eMERGE. The "PGS effect size" column contains the effect size (differences in phenotypic standard deviation, SD), or odds ratio for CAD, per SD of PGS, accounting for covariates. "se": standard error of the beta coefficient. The "PGS p-value" column contains the two-sided p-values which were not adjusted for multiple comparisons. Effect sizes and p-values were estimated from linear regression models for quantitative traits and logistic regression models for coronary artery disease (CAD). "Relative accuracy" shows the ratio of predictive accuracy (incremental AUC for CAD, or incremental r2 for continuous traits) in G&H to that in eMERGE. PGS ID, PGS Name and the publication PMID are from the PGS Catalog (http://www.pgscatalog.org). PGSs for CAD and BMI were developed and optimised in individuals of European ancestry (EUR) or South Asian ancestry (SAS), and PGSs for lipids and BP contained Genome-wide significant (GWS) variants. More information such as ancestry distribution of the GWAS discovery cohorts and PGS training cohorts are provided in the PGS Catalog.

File Name: Supplementary Data 6
Description: Characteristics of G&H participants. The values shown are mean (standard deviation) unless otherwise indicated. *For lipid traits and blood pressure, the table summarises the latest value measured within the past five years without adjusting for medications.

File Name: Supplementary Data 7
Description: SNP heritability of cardiometabolic traits in G&H and eMERGE, estimated with GCTA. We present results using all SNPs with INFO >0.9 and MAF >0.01 available in each cohort separately, and with the intersection of these SNP sets (N=2,228,506) in both cohorts. We performed a two-sample z test to compare the heritability estimates between the two cohorts. One-sided, unadjusted p-values are reported.

File Name: Supplementary Data 8
Description: Cardiometabolic loci transferable between Europeans and British Pakistani and Bangladeshis. *Lead SNP identified from discovery GWAS was either absent or not significant in G&H, thus summary stats are presented for the best proxy in the credible set. We used SAIGE to perform GWAS using linear mixed models and the p-values reported here are two-sided and not adjusted for multiple comparisons.

File Name: Supplementary Data 9
Description: Transferability of loci for cardiometabolic phenotypes from European ancestry discovery GWAS to European ancestry individuals in eMERGE using the power-adjusted transferability (PAT) ratio. For SBP and DBP, power was calculated with effect size in UK Biobank European-ancestry individuals by Neale's group where normalised BP values were used. In eMERGE, medication data were not available thus we used the highest measurements for LDL-C, SBP, and DBP. CAD was defined based on ICD10 codes only. P-values are from one-sided binomial tests and are not adjusted for multiple comparisons.

File Name: Supplementary Data 10
Description: Loci associated in published European-ancestry GWAS that were not transferable to British Pakistanis and Bangladeshis despite statistical power > 0.8. We used SAIGE to perform GWAS using linear mixed models and the p-values reported here are two-sided and not adjusted for multiple comparisons. * Tag SNP: rs7499892, r2=1.

File Name: Supplementary Data 11
Description: Results from trans-ancestry colocalisation with JLIM to assess sharing of causal variants. The "p-value" columns from the "UKBB EUR" and "G&H BPB" contain two-sided, unadjusted p-values from GWAS. We used SAIGE to perform GWAS in G&H using linear mixed models. "p_JLIM" indicates the posterior probability of significant colocalisation reported by JLIM.

File Name: Supplementary Data 12
Description: Details and predictive accuracy of PGSs constructed using the clumping and p-value thresholding (C+T) method. We performed 10-fold cross validation and repeated 100 times. For each left-out fold, we calculated the prediction accuracy for PGS constructed using parameters that were selected in the other nine folds. The parameters for the most frequently selected PGS are shown. We calculated the average predictive accuracy across 10 folds and 100 repeats. The "relative accuracy" column shows the ratio of mean PGS accuracy (incremental AUC for CAD, or incremental r2 for quantitative traits) in G&H to that in eMERGE. "CI": confidence interval.

File Name: Supplementary Data 13
Description: Performance of different models used to predict CAD risk in different subsets of G&H. The concordance index (C-index) and its standard error (se) were calculated for the following models: (1) age and sex, (2) PGS for CAD, age, and sex, (3) QRISK3, and (4) the integrated score combining QRISK3 and

the CAD PGS. *Sex was not adjusted for in the age-by-sex subgroups. Continuous net reclassification index (NRI) and categorical NRI in all samples, cases, and controls are shown. This table also contains the number and proportion of individuals with QRISK3 ≥10% in each group (QRISK3 predictions), the number and proportion of individuals with the integrated risk score ≥10% (IRT predictions), the number and proportion of individuals that were re-classified to the high-risk group by the integrated risk score (IRT up), the number and proportion of cases that were re-classified as high-risk individuals (IRT up in cases), the number and proportion of individuals that were re-classified to the low-risk group (IRT down), and the number and proportion of controls that were re-classified as low-risk individuals (IRT down in controls). Top: standard Method used in the manuscript. Bottom: sensitivity analysis where data measured at a later point were also included in the QRISK3 analysis (Supplementary Methods).

File Name: Supplementary Data 14
Description: MR Sensitivity analyses. P-values (two sided, not adjusted for multiple comparisons) and effect sizes for inverse-variance weighted (IVW), weighted median and weighted mode models, MR Egger intercept, Cochran's Q statistic and MR-PRESSO.

File Name: Supplementary Data 15
Description: Multivariable MR. Direct effect size estimates of HDL-C, LDL-C and TG on the risk of CAD. P-values are two-sided and not adjusted for multiple comparisons. We applied the analysis using the multivariable MR (MVMR) setting with the TwoSampleMR R package.
