# Supplementary material for: Transferability of genetic loci and polygenic scores for cardiometabolic traits in British Pakistani and Bangladeshi individuals
Source: Nat Commun. 2022 Aug 9;13:4664. doi: 10.1038/s41467-022-32095-5 (PMC9363492; doi:10.1038/s41467-022-32095-5)
Supplement: Supplementary file 5 — Reporting Summary [file 41467_2022_32095_MOESM5_ESM.pdf]

nature portfolio

Corresponding author(s): Karoline Kuchenbäcker
Last updated by author(s): Jun 20, 2022

Reporting Summary

Nature Portfolio wishes to improve the reproducibility of the work that we publish. This form provides structure for consistency and transparency in reporting. For further information on Nature Portfolio policies, see our Editorial Policies and the Editorial Policy Checklist.

Statistics

For all statistical analyses, confirm that the following items are present in the figure legend, table legend, main text, or Methods section.

n/a	Confirmed	
☐	☒	The exact sample size (*n*) for each experimental group/condition, given as a discrete number and unit of measurement
☐	☒	A statement on whether measurements were taken from distinct samples or whether the same sample was measured repeatedly
☐	☒	The statistical test(s) used AND whether they are one- or two-sided<br>*Only common tests should be described solely by name; describe more complex techniques in the Methods section.*
☐	☒	A description of all covariates tested
☐	☒	A description of any assumptions or corrections, such as tests of normality and adjustment for multiple comparisons
☐	☒	A full description of the statistical parameters including central tendency (e.g. means) or other basic estimates (e.g. regression coefficient) AND variation (e.g. standard deviation) or associated estimates of uncertainty (e.g. confidence intervals)
☐	☒	For null hypothesis testing, the test statistic (e.g. *F*, *t*, *r*) with confidence intervals, effect sizes, degrees of freedom and *P* value noted<br>*Give P values as exact values whenever suitable.*
☒	☐	For Bayesian analysis, information on the choice of priors and Markov chain Monte Carlo settings
☒	☐	For hierarchical and complex designs, identification of the appropriate level for tests and full reporting of outcomes
☐	☒	Estimates of effect sizes (e.g. Cohen's *d*, Pearson's *r*), indicating how they were calculated

Our web collection on statistics for biologists contains articles on many of the points above.

Software and code

Policy information about availability of computer code

Data collection	Genes & Health participants were genotyped on the Illumina Infinium Global Screening Array v3 with additional multi-disease variants. Quality control of genotype data was performed using Illumina's GenomeStudio and plink v1.9. No custom software was used.
Data analysis	Imputation of genotype data were performed using the Michigan Imputation Server, where Eagle (v 2.4) was used for phasing and Minimac (v 4) was used for imputation. GWAS was performed using the SAIGE (v 0.39) software. QC of GWAS was performed using the EasyQC (v 23.8) package. Heritability was estimated using GCTA-GREML (gcta v 1.93.0b). Trans-ancestry genetic correlation was estimated using Popcorn (v 0.9.9). Trans-ancestry colocalisation analysis was performed using TAColoc (https://github.com/KarolineKuchenbaecker/TEColoc). LocusZoom (v 0.12) was used to create regional association plots. Clumping and P-value thresholding PGS were calculated using PRSice2 (v 2.2.11). Identification of related individuals was performed using KING (v 2.2.4). Principle component analysis was performed using smartpca from EIGENSOFT (v 7.2.1). Identification of European-ancestry individuals in eMERGE was performed using the umap R package (v 2.6.0). plink 2.0 was used to calculate polygenic scores downloaded from the PGS Catalog based on imputed dosage data. The area under the receiver operating characteristic curve (AUC) of PGS was estimated using the pROC R package (v 1.16.2). QRISK3 scores were calculated using the QRISK3 R package (v 0.3.0) and multiple imputation of QRISK3 variables was performed using the mice R package (v 3.13.0). Cox regression was performed using the R package survival (v 3.2-7). Mendelian randomisation analysis was performed using the TwoSampleMR R package. PRS-CSx (v 1.0.0) was used to calculate trans-ancestry polygenic scores.<br><br>Custom codes can be found at https://github.com/Nsallah1/GH_Manuscript and https://github.com/QinqinHuang/GnH28k_polygenic_scores.

For manuscripts utilizing custom algorithms or software that are central to the research but not yet described in published literature, software must be made available to editors and reviewers. We strongly encourage code deposition in a community repository (e.g. GitHub). See the Nature Portfolio guidelines for submitting code & software for further information.

Data

Policy information about availability of data

All manuscripts must include a data availability statement. This statement should provide the following information, where applicable:

- Accession codes, unique identifiers, or web links for publicly available datasets
- A description of any restrictions on data availability
- For clinical datasets or third party data, please ensure that the statement adheres to our policy

Genes & Heath imputed genotype data (GRCh 37) have been deposited in EGA under study accession number: EGAS00001005373 (https://ega-archive.org/datasets/EGAD00001007815). The electronic health records from Genes & Health are available under restricted access for bona fide research; researchers wishing to access them should apply to the G&H Executive (www.genesandhealth.org/research/scientists-using-genes-health-scientific-research). GWAS summary statistics generated in Genes & Health are available at www.genesandhealth.org/research/scientific-data-downloads. The transferable loci generated in this study are provided in the Supplementary Data file. Publicly available GWAS summary statistics that were used in this study (Supplementary Data 4) are available via the CARDIoGRAMplusC4D Consortium (http://www.cardiogramplusc4d.org), GIANT (https://portals.broadinstitute.org/collaboration/giant/index.php/Main_Page), GLGC (http://csg.sph.umich.edu/willer/public/lipids2017/), and GWAS Atlas (https://atlas.ctglab.nl/traitDB/). SNPs and the weights for polygenic risk scores are available in the PGS Catalog (www.pgscatalog.org) and score IDs are provided in Supplementary Data 5.

Field-specific reporting

Please select the one below that is the best fit for your research. If you are not sure, read the appropriate sections before making your selection.

☒ Life sciences ☐ Behavioural & social sciences ☐ Ecological, evolutionary & environmental sciences

For a reference copy of the document with all sections, see nature.com/documents/nr-reporting-summary-flat.pdf

Life sciences study design

All studies must disclose on these points even when the disclosure is negative.

Sample size	Sample size was determined by the largest available data release when the analysis was performed.
Data exclusions	Genes & Health: Samples with low call rate (<0.99) were excluded. Individuals who did not have Bangladeshi or Pakistani ancestry from either the genetic data or self-reported data were excluded.<br>eMERGE: Samples with non-European ancestry were excluded.
Replication	Our study assessed how many genetic associations identified in primarily European-ancestry populations replicated in South Asian ancestry individuals from the Genes & Health cohort at nominal significance threshold. For loci with sufficient power to replicate, we observed that the majority of the genetic loci were replicated in Genes & Health and the one-sided p-values for the power-adjusted transferability (PAT) ratio were not significant after adjusting for multiple comparisons.<br>This finding itself was not replicated in another large South Asian ancestry cohort with relevant phenotypic data, due to the fact that such cohorts are not easily available to our knowledge.
Randomization	Randomisation was not required for the genetic analyses we performed in the paper. In all statistical analyses, we controlled for the following covariates: sex (unless the analyses were performed in males and females separately), age, and first 10 genetic principal components which capture the population structure.
Blinding	All samples were deidentified. Genotyping and phenotypic data curation were performed independently.

Reporting for specific materials, systems and methods

We require information from authors about some types of materials, experimental systems and methods used in many studies. Here, indicate whether each material, system or method listed is relevant to your study. If you are not sure if a list item applies to your research, read the appropriate section before selecting a response.

Materials & experimental systems

n/a	Involved in the study	
☒	☐	Antibodies
☒	☐	Eukaryotic cell lines
☒	☐	Palaeontology and archaeology
☒	☐	Animals and other organisms
☐	☒	Human research participants
☒	☐	Clinical data
☒	☐	Dual use research of concern

Methods

n/a	Involved in the study	
☒	☐	ChIP-seq
☒	☐	Flow cytometry
☒	☐	MRI-based neuroimaging

Human research participants

Policy information about studies involving human research participants

Population characteristics	Description of the Genes & Health cohort and population characteristics is available in the first paragraph of the Results section, first paragraph of the Methods section, and Supplementary Table6.<br>Detailed cohort profile is available in the published paper by Finer et al. 2020 (reference 13: https://doi.org/10.1093/ije/dyz174).
Recruitment	Recruitment of the Genes & Health cohort is described in the cohort profile paper by Finer et al. 2020. In short, Genes & Health is a community-based cohort of British Pakistani and Bangladeshi individuals recruited primarily in East London. The cohort is broadly representative of the background population with regard to age, but slightly over-sampled females and those with medical problems since two-thirds of people were recruited in healthcare settings such as GP surgeries.
Ethics oversight	The study was approved by the London South East NRES Committee of the Health Research Authority (14/LO/1240).

Note that full information on the approval of the study protocol must also be provided in the manuscript.
